# High femoral offset as a risk factor for aseptic femoral component loosening in cementless primary total hip arthroplasty

**DOI:** 10.1007/s00264-024-06116-5

**Published:** 2024-02-23

**Authors:** Lukas Jud, Nico Rüedi, Dimitris Dimitriou, Armando Hoch, Patrick O. Zingg

**Affiliations:** Balgrist University Hospital, Department of Orthopedics, University of Zurich, Forchstrasse 340, 8008 Zürich, Switzerland

**Keywords:** Aseptic loosening, Femoral offset, Femoral head length, Primary THA, Odds ratio

## Abstract

**Purpose:**

Lateralized stems in primary cementless total hip arthroplasty (THA) showed to be associated with aseptic femoral loosening. However, femoral head length also affects femoral offset but was not considered so far. This study analyzed the impact of high femoral offset (hFO) combinations, formed by lateralized stems or large femoral head lengths, on aseptic femoral component loosening.

**Methods:**

Retrospective cohort study was performed including all patients that underwent primary cementless THA at our institution between July 2004 and December 2016. Patients were screened for aseptic femoral component loosening and grouped in aseptic loosening (AL) and non-aseptic loosening (nAL) group. Medical records were screened; implant details were noted and classified in hFO and standard femoral offset (sFO) combinations. Supposed risk factors for aseptic loosening were analyzed.

**Results:**

Two thousand four hundred fifty-nine THA could be included, containing 14 THA (0.6%) with aseptic femoral component loosening. The AL group contained 11 hFO combinations (78.6%), whereas in the nAL group, 1315 hFO combinations (53.8%) were used. Subgroup analysis showed significant difference between two groups for hFO combinations (*p* = 0.014), age (*p* = 0.002), NSAR (*p* = 0.001), and bilateral THA on same day (*p* = 0.001). The multiple logistic regression analysis showed that hFO combination was the only variable for increased probability of aseptic loosening (OR, 3.7; *p* = 0.04).

**Conclusion:**

High femoral offset combinations, formed by lateralized stems or large femoral head lengths in our collective of standard straight stems implanted by an anterior approach, show a 3.7-fold increased probability for aseptic femoral component loosening. Adjustment of the postoperative protocol may be considered in these cases to ensure proper stem ingrowth.

## Introduction

In total hip arthroplasty (THA), restoration of patients’ native global offset is beneficial regarding patient-reported outcomes and implant survival [1–4]. While an increased offset bears the risk of increased postoperative lateral hip pain, a reduced offset potentially results in alteration of the gait cycle, increased joint reaction forces, and THA instability [5–9]. The native global offset can vary essentially between individuals, wherefore in cases with high native offsets, lateralized implants may be needed for appropriate restoration of their anatomy [7, 10–13]. However, as high femoral offset stems showed to be associated with increased micro motion on the implant-bone interface, there exist concerns about the use of lateralized femoral implants due to increased reported rates of aseptic loosening compared to non-lateralized standard stems [12, 14–16]. Besides the use of a lateralized stem design, the use of a larger femoral head length affects femoral offset likewise. We observed in cementless THA from different manufacturers that combination of standard femoral stems and femoral head lengths of the size “large” or “extra-large” shows equivalent or larger femoral offset than the combination of a lateralized stem and a femoral head length of the size “small.” Previous studies on cementless THA focused only on lateralized stems as a risk factor for aseptic loosening, not considering the femoral head lengths [14, 15]. Therefore, a retrospective cohort study was performed including all patients that underwent primary cementless THA using an anterior approach from July 2004 to December 2016 at our institution. Femoral offset combinations were determined, and patients grouped regarding the event of aseptic femoral component loosening. Furthermore, patients were screened for other supposed risk factors for aseptic loosening (i.e., non-steroidal antirheumatics (NSAR), age, nicotine) [15, 17–19]. We hypothesize that patients in whom an implant combination with high femoral offset was used show higher revision rates for aseptic loosening than patients with a combination of standard femoral offset.

## Materials and methods

**Fig. 1:**
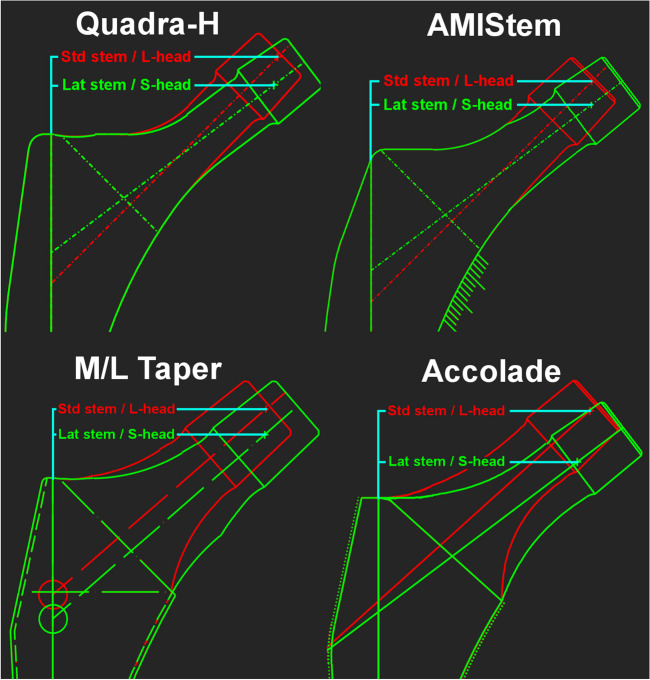
Equivalent or higher femoral offset (turquoise) in standard stems (red) using femoral head lengths of the size large (L) compared to lateralized stems (green) using femoral head lengths of the size small (S), shown for Quadra-H stem and AMIStem (Medacta, Castel San Pietro, Switzerland), M/L Taper stem (Zimmer, Warsaw, IN, USA), and Accolade stem (Stryker, Mahwah, NJ, USA). Std, standard; Lat, lateral

**Fig. 2:**
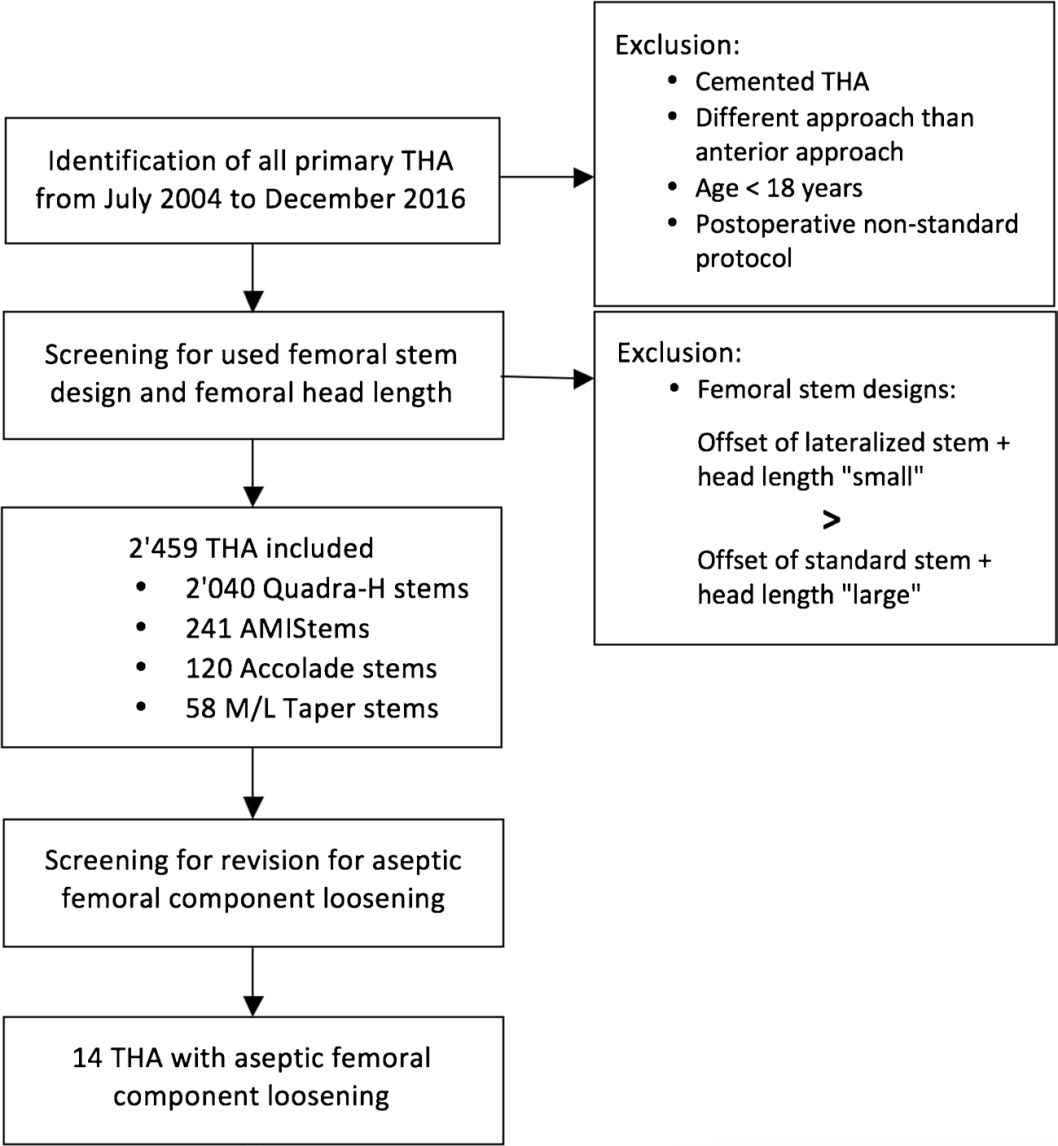
Flow chart of the inclusion process. THA, total hip arthroplasty

**Fig. 3:**
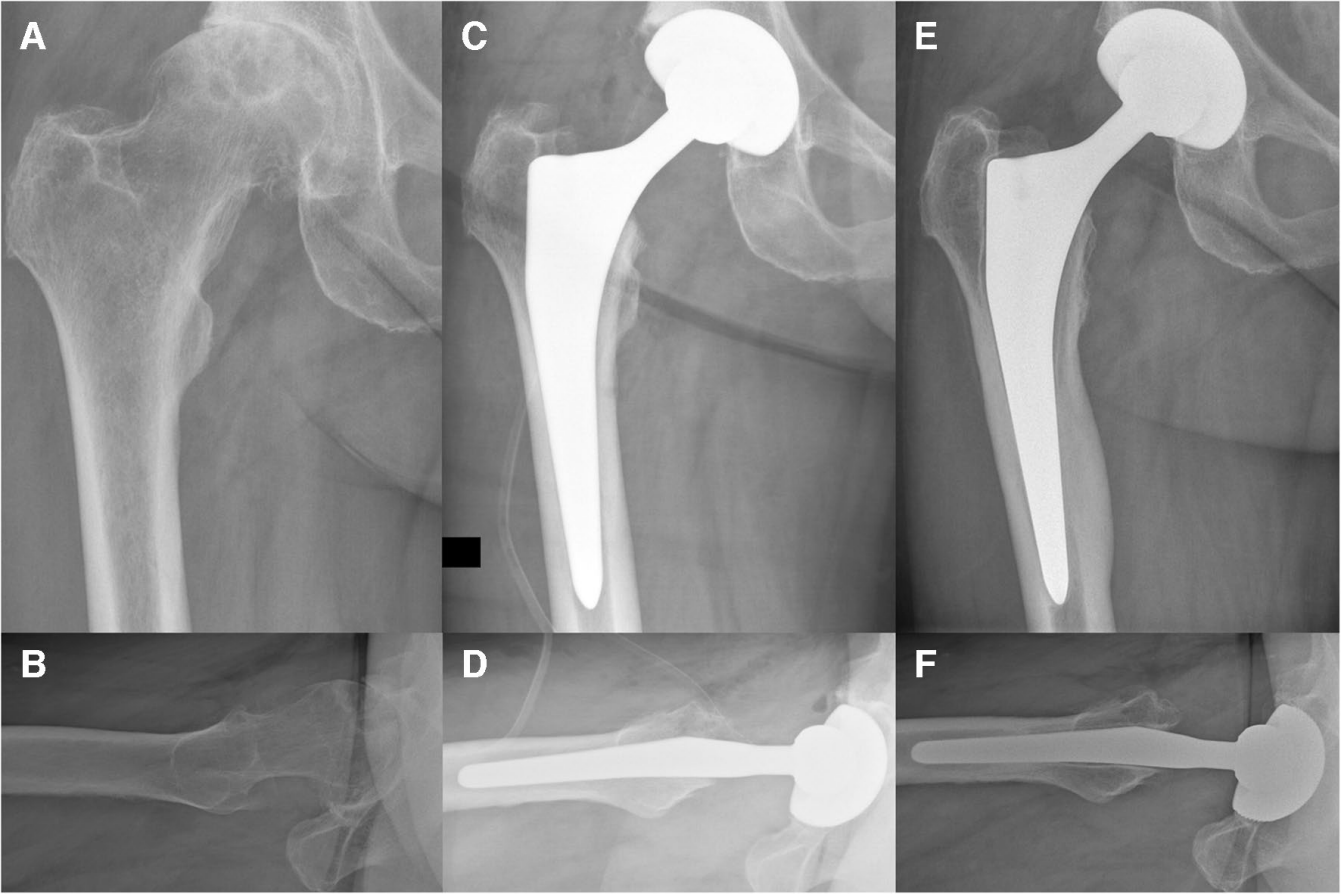
Conventional radiographs of a patient with aseptic femoral component loosening. **A**, **B** Preoperative radiographs. **C**, **D** The direct postoperative radiographs, whereas **E**, **F** show the loose femoral component 3 years and 9 months postoperative

A retrospective cohort study was performed. All patients that underwent primary cementless THA by an anterior approach between July 2004 and December 2016 at our institution were identified. Cementless THA and the use of the anterior approach are the standard for primary THA at our institution since the start of the inclusion period. The anterior approach is used whenever appropriate, and a different approach is only applied in case of reasonable doubts. In case of concerns of the respective bone quality, as in clear Dorr type C femurs, we consider the possibility of a cemented THA. Only adult patients (i.e., ≥ 18 years) and patients referred to a postoperative standard protocol were included. The standard protocol allowed immediate postoperative full weight bearing with free range of motion but using crutches for two weeks and avoidance of external rotation in extension for three months. In all cases, two-dimensional THA planning on standardized antero-posterior pelvic radiographs, with 15° of internal rotation of the lower extremity, was performed. Position of the acetabular component was planned to the acetabular fossa, and patient’s native global offset was reconstructed using the respective femoral stem and head length. In case of severe deformity or inability of correct internal rotation of the affected lower extremity, the healthy contralateral side was used for reference of offset restoration. Regarding the implants of different manufacturers, combinations of standard femoral stems and femoral head lengths of the size “large” or “extra-large” show equivalent or larger femoral offset than the combination of a lateralized stem of the same size and a femoral head length of the size “small.” This can be observed for the following implants, which have been used in our institution during the inclusion period: Quadra-H stem and AMIStem (Medacta, Castel San Pietro, Switzerland), M/L Taper stem (Zimmer, Warsaw, IN, USA), and Accolade stem (Stryker, Mahwah, NJ, USA) (Fig. 1). Regarding head lengths, for Quadra-H stem, AMIStem, and the M/L Taper stem, a head length small corresponded to a size − 3.5 mm, “medium” to 0 mm, large to + 3.5 mm, and extra-large to + 7 mm. Regarding the Accolade stem, the head length small corresponded to a size − 4 mm, medium to 0 mm, large to + 4 mm, and extra-large to + 8 mm. All of the used implants correspond to standard straight stem designs. Therefore, only THA using these types of implants were included. A flow chart of the inclusion is visualized in Fig. 2. Implant combinations using a lateralized stem and/or a femoral head length of the size large or extra-large were handled as high femoral offset (hFO) combinations, whereas implant combinations using a standard stem and a femoral head length of the size small or medium were handled as standard femoral offset (sFO) combinations. Individual implant combinations were chosen following the preoperative standardized THA planning. All patients were screened for the event of revision for aseptic femoral component loosening, between July 2004 and December 2022, and accordingly grouped in an aseptic loosening (AL) group and a non-aseptic loosening (nAL) group. Aseptic loosening was diagnosed using clinical examination, conventional radiographs (Fig. 3), and eventually magnetic resonance imaging. In all cases, the loose femoral component was confirmed intraoperatively by loose extraction of the implant. Postoperative offset restoration and early stem subsidence at three months postoperatively were controlled in the AL group. Besides noting the implant details, medical records were screened for postoperative NSAR, age, and nicotine abuse, presenting other assumed risk factors for aseptic loosening [15, 17–19]. Furthermore, details were noted for THA indication (primary osteoarthritis or other reason), gender, side, height, weight, and bilateral THA on the same day. Preoperative Dorr classification was evaluated in all patients.

The local ethical committee approved this study.

### Statistical analysis

Descriptive data for categorical variables were summarized with frequencies, while continuous parameters were described by averages, ranges, and standard deviations. Comparisons among categorical data (gender, diagnosis, side, lateral stem, NSAR) were evaluated using Pearson’s chi-squared test. All continuous parameters (age, BMI) were tested with the Kolmogorov-Smirnov test for normality, and a two-tailed unpaired *t* test was then used to compare this data between patients with and without aseptic loosening of the femoral stem. A univariable logistic regression analysis was performed to identify independent risk factors for stem loosening. All independent risk factors were thereafter analyzed via multivariate logistic regression analysis, and regression coefficients and odds ratios (OR) were calculated. A paired *t* test was performed to evaluate potential differences of preoperative and postoperative femoral offset in the AL group. All the statistical analyses were performed using SPSS version 23 software (SPSS Inc., Chicago, IL).

## Results

A total of 2459 THA in 2114 patients (1052 women and 1193 left sides) could be included, containing 14 THA (0.6%) with aseptic femoral component loosening in 12 patients (Table 1). In the AL group, revision surgery for aseptic femoral component loosening was performed in mean after 48 ± 22 months (range 17 to 84 months). Overall, mean age was 63.8 ± 12.7 years (range 18 to 92 years) and mean body mass index (BMI) was 26.8 ± 5.2 kg/m^2^ (range 13.2 to 58.5 kg/m^2^). Two thousand forty Quadra-H stems (Medacta, Castel San Pietro, Switzerland), 241 AMIStems (Medacta, Castel San Pietro, Switzerland), 120 Accolade stems (Stryker, Mahwah, NJ, USA), and 58 M/L Taper stems (Zimmer, Warsaw, IN, USA) were used. The AL group contained ten (0.5%) Quadra-H stems (Medacta, Castel San Pietro, Switzerland), two (0.8%) AMIStems (Medacta, Castel San Pietro, Switzerland), one (0.8%) Accolade stem (Stryker, Mahwah, NJ, USA), and one (1.7%) M/L Taper stem (Zimmer, Warsaw, IN, USA). A total of 1326 hFO combinations were used. In the AL group, hFO combinations were used in 78.6% of THA (11/14), whereas in the nAL group, hFO combinations were used in 53.8% of THA (1315/2445). Control for the offset restoration in the AL group showed no significant changes of offset with a mean postoperative deviation of − 0.4 ± 1.0 mm (range − 2 to 2 mm). Control for early stem subsidence in the AL group at three months postoperatively showed no significant changes with a mean stem subsidence of 0.5 ± 0.5 mm (range 0 to 1 mm). The subgroup analysis showed statistically significant differences between the two groups for hFO combinations (*p* = 0.014), age (*p* = 0.002), NSAR (*p* = 0.001), and bilateral THA on same day (*p* = 0.001). An overview of the subgroup analysis is given in Table 2. The multiple logistic regression analysis showed age (OR, 0.93; 95% CI, 0.869–0.968; *p* = 0.001) and NSAR (OR, 0.03; 95% CI, 0.01–0.1; *p* < 0.001) to be associated with decreased odds for femoral component loosening, whereas the use of a hFO combination showed to be associated with increased odds for aseptic loosening (OR, 3.7; 95% CI, 1.01–13.8; *p* = 0.04). All other analyzed factors showed not to be independent risk factors for aseptic loosening in the multiple logistic regression analysis.

**Table 1:** Detailed overview of aseptic loosening group

No.	Age (years)	Gender	BMI (kg/m^2^)	Diagnosis	Implant type	Stem design	Stem size	Head length	hFO	Postop offset-diff. (mm)	Stem subsidence (mm)	Nicotine	NSAR	Bilateral THA same day	Dorr- classification
1	76	m	26.4	Primary OA	Accolade	Standard	3	Large	Yes	− 2	0	No	Yes	no	A
2	56	w	25.7	Primary OA	M/L Taper	Lateralized	7.5	Medium	Yes	0	1	No	Yes	no	A
3	69	m	28.4	Primary OA	Quadra-H	Lateralized	7	Large	Yes	1	0	No	Yes	no	A
4	59	m	26.8	Primary OA	Quadra-H	Lateralized	4	Large	Yes	− 1	1	No	Yes	no	A
5	35	m	27.7	Osteonecrosis	Quadra-H	Standard	1	Large	Yes	1	0	Yes	No	no	A
6	47	w	25.5	Primary OA	Quadra-H	Standard	2	Small	No	− 1	0	No	No	yes	A
7	47	w	25.5	Primary OA	Quadra-H	Standard	2	Small	No	2	0	No	No	yes	A
8	53	m	34.4	Primary OA	AMIStem	Standard	6	Large	Yes	0	1	No	Yes	no	A
9	40	m	29.0	Primary OA	Quadra-H	Lateralized	2	Large	Yes	− 1	1	No	No	yes	A
10	40	m	29.0	Primary OA	Quadra-H	Lateralized	2	Large	Yes	− 1	1	No	No	yes	A
11	60	m	29.7	Primary OA	AMIStem	Lateralized	3	Medium	Yes	− 1	0	No	Yes	no	A
12	45	w	24.7	Primary OA	Quadra-H	Lateralized	4	Small	Yes	− 1	1	Yes	No	no	A
13	66	w	27.8	Primary OA	Quadra-H	Lateralized	3	Large	Yes	0	1	No	Yes	no	A
14	55	w	26.2	Primary OA	Quadra-H	Standard	3	Small	No	− 1	0	No	Yes	no	A

*BMI* body mass index, *hFO* high femoral offset combination, *diff* difference, *NSAR* non-steroidal antirheumatics, *THA* total hip arthroplasty

**Table 2:** Subgroup analysis

	AL group (*n* = 14)	nAL group (*n* = 2445)	
Age	53.4 ± 11.9 (35 to 76) years	63.8 ± 12.7 (18 to 92) years	*p* = 0.002
Gender			
*Female*	6	1211	n.s.
*Male*	8	1234	
BMI	27.6 ± 2.5 (24.7 to 34.4) kg/m^2^	26.8 ± 5.2 (13.0 to 58.0) kg/m^2^	n.s.
Side			
*Left*	8	1185	n.s.
*Right*	6	1260	
Diagnosis			
*Primary OA*	13	2029	n.s.
*Others*	1	416	
Nicotine abuse	2	670	n.s.
NSAR	8	2390	*p* = 0.001
Bilateral THA on same day	4	126	*p* = 0.001
Femoral offset			
*hFO*	11	1304	*p* = 0.014
*sFO*	3	1141	
Dorr classification			
*A*	14	2037	n.s.
*B*	0	406	
*C*	0	2	

*AL* aseptic loosening group, *nAL* non-aseptic loosening group, *BMI* body mass index, *OA* osteoarthritis, *NSAR* non-steroidal antirheumatics, *THA* total hip arthroplasty, *hFO* high femoral offset combination, *sFO* standard femoral offset combination

## Discussion

The most important finding of the present study was that the use of a hFO combination, formed either by lateralized stems or standard stems combined with femoral head lengths of the size large or extra-large, was associated with increased odds for aseptic femoral component loosening. Therefore, the hypothesis of this study could be confirmed. Increased risk for aseptic femoral component loosening in lateralized stems has already been described in the literature. In a cohort of 807 primary cementless THA, including 280 lateralized stems, Cantin et al. [14] showed significant increased risk for aseptic loosening in lateralized stems. They identified five cases with aseptic femoral component loosening in their cohort, all in patients with lateralized stems. However, femoral head lengths were not considered in their study; grouping in regards of femoral offset was only performed by femoral stem design. Another study by Courtin et al. [15] investigated the occurrence of symptomatic radiological changes in lateralized femoral stems, likewise not considering femoral head length. In their cohort of 172 cases with lateralized stems in primary cementless THA, they identified a revision rate for aseptic loosening of 4.1% at mid-term follow-up and therefore higher than compared to previous literature with revision rates between 1.3 and 2.7% [20], and also as the reported overall revision rate of 0.6% in our study. So far, the increased rates of aseptic loosening in lateralized implants are explained by the accelerated torsional stress and micro motion on the implant-bone interface [12, 16, 21]. However, in regards of the principle of the lever arm, the accelerated torsional stress and subsequent micro motions are affected by the total femoral offset, including the femoral head length beside the stem design. Hence, only considering lateralized implants when investigating high femoral offsets as a risk factor for aseptic loosening would probably underestimate the real impact of increased femoral offset combinations. The current study is the first considering the increase of femoral offset by the femoral head length and the impact of overall high femoral offset combinations on aseptic femoral component loosening. The multiple logistic regression analysis showed a 3.7-fold increased odd for aseptic femoral component loosening when using a hFO combination. This may indicate a forward-thinking preoperative THA planning, using lateralized implants only in cases in which offset restoration with standard stems is not possible and trying to avoid excessive femoral head lengths. In cases in which the avoidance of hFO combinations seem not reasonable, a more restrictive postoperative protocol probably may allow proper stem ingrowth and therefore diminish the risk for aseptic loosening, as partial weight bearing is known to result in less stem subsidence [22].

Beside high femoral offset, the postoperative usage of NSAR is also discussed as a reason for aseptic loosening, due to drug-induced bone remodeling effects [18, 19]. However, in our cohort, patients using postoperative NSAR showed a decreased probability for aseptic femoral component loosening. Likewise, higher age showed to be associated with decreased probability for aseptic loosening in our study, matching the results of previous studies [15]. A possible explanation for the decreased probability for aseptic loosening in higher age might be the presumed lower activity in the early postoperative period in this population. Regarding the nicotine consumption, increased risk for aseptic loosening has been shown in proximal mega-prosthetic femoral replacement [17]. However, nicotine abuse showed no increased probability for aseptic loosening in our cohort. Furthermore, concerns may exist in cementless THA on stem ingrowth in biological compromised proximal femur, although this could not yet be confirmed in the current literature [23]. Likewise, a higher Dorr classification showed no increased odds for aseptic loosening in our cohort.

The present study should be interpreted in light of its potential limitations. First, diagnosis for aseptic femoral component loosening was only detected if patients have undergone revision surgery at our institution. If a patient had revision at a different institution, this patient could not be detected and could not be grouped to the AL group. However, as a tertiary university hospital, containing a specialized hip unit, no patient was referred to a different institution from our site. Therefore, the number of patients that specifically decided to undergo revision surgery at a different hospital should be small. Another limitation is that the control for offset restoration was performed using plane antero-posterior radiographs instead of computed tomography scans [24]. Furthermore, offset restoration was only controlled in the AL group; in the nAL group, postoperative offset restoration was not controlled. However, standardized THA planning was performed in every case and in a way to restore the native global offset. The accurate offset restoration is reflected in the demonstrated small postoperative deviation in the AL group and why it was supposed that similar offset restoration was achieved in the remaining patients. Nonetheless, a possible deviation in offset restoration in the nAL group would have had no influence on the event of aseptic loosening in this group. Another limitation that should be mentioned is that only patients undergoing THA using an anterior approach were included in this study and the anterior approach is known to be a risk factor for stem undersizing [25], resulting in a possible early stem subsidence. However, we were not able to detect any relevant stem subsidence in the AL group. Therefore, using a different surgical approach than the anterior approach should not interfere the results observed in this study. Finally, the limited number of cases with aseptic loosening has to be mentioned and wherefore this study is underpowered regarding the number of analyzed risk factors.

## Conclusion

High femoral offset combinations, formed by lateralized stems or large femoral head lengths in our collective of standard straight stems implanted by an anterior approach, show a 3.7-fold increased probability for aseptic femoral component loosening. Adjustment of the postoperative protocol may be considered in these cases to ensure proper stem ingrowth.
